# The Transaxillary Approach *via* Prosthetic Conduit for Transcatheter Aortic Valve Replacement With the New-Generation Balloon-Expandable Valves in Patients With Severe Peripheral Artery Disease

**DOI:** 10.3389/fcvm.2021.795263

**Published:** 2022-01-13

**Authors:** Alexander Lind, Alina Zubarevich, Arjang Ruhparwar, Matthias Totzeck, Rolf Alexander Jánosi, Tienush Rassaf, Fadi Al-Rashid

**Affiliations:** ^1^Department of Cardiology and Vascular Medicine, West-German Heart and Vascular Center Essen, University of Duisburg-Essen, Essen, Germany; ^2^Department of Heart Surgery, West-German Heart and Vascular Center Essen, University of Duisburg-Essen, Essen, Germany

**Keywords:** TAVR, axillary access, conduit, prosthetic, Dacron, balloon-expandable prosthesis, percutaneous-methods

## Abstract

**Background:** The left subclavian artery (LSA) is an infrequently used alternative access route for patients with severe peripheral artery disease (PAD) in patients who underwent transcatheter aortic valve replacement (TAVR). We report a new endovascular approach for TAVR combining an axillary prosthetic conduit-based access technique with new-generation balloon-expandable TAVR prostheses.

**Methods and Results:** Between January 2020 and December 2020, 251 patients underwent TAVR at the West German Heart and Vascular Center. Of these, 10 patients (3.9%) were deemed to be treated optimally by direct surgical exposure of the left or right axillary artery *via* a surgically adapted prosthetic conduit. All procedures were performed under general anesthesia. One procedural stroke occurred due to severe calcification of the aortic arch. No specific complications of the subclavian access site (vessel rupture, vertebral, or internal mammary ischemia) were reported. Two minor bleedings from the access site could be treated conservatively. No surgical revision was necessary.

**Conclusion:** The axillary prosthetic conduit-based access technique using new-generation balloon-expandable valves allows safe and successful TAVR in a subgroup of patients with a high risk of procedural complications due to severe peripheral vascular disease. Considering the increasing number of patients referred for TAVR, this approach could represent an alternative for patients with limited access sites.

## Introduction

Transcatheter aortic valve replacement (TAVR) continues to expand rapidly as a less invasive option for the treatment of severe aortic stenosis (AS) in patients considered at intermediate or high risk during surgical aortic valve replacement (1, 2). Delivery systems have evolved, corresponding sheath sizes have also diminished to facilitate higher rates of transfemoral (TF-) TAVR. Therefore, TF access is the state-of-the-art access route for TAVR procedures with documented low periprocedural complications enabling early mobilization and discharge (3). However, a growing number of patients requiring TAVR may have femoral access issues, usually related to severe peripheral artery disease (PAD), small iliofemoral arteries, and comorbidities, such as hostile aortoiliac segment occlusive disease (3, 4). Initially, the transapical (TA) and transaortic (TAo) approaches were used whenever a TF approach was not anatomically feasible. However, the use of these non-arterial accesses was associated with worse outcomes, partially because of the need for thoracotomy (4–6). Due to the disappointing outcomes associated with these more traditional alternative access routes, alternative access sites, including transaxillary (TAx), trans-subclavian (TS), transcarotid, and transcaval access, have been developed (7–10). The TAx approach is considered the second option in many centers when TF-TAVR is not feasible. Within the more popular TAx and TS approaches, procedural techniques vary widely and most of the interventions using the TAx access have been performed with self-expanding valve platforms considering the necessity of assembling the balloon-expandable valve system in the ascending aorta (11–14).

Vessel access is gained either *via* open surgical access through an infraclavicular incision and direct insertion of a large-bore sheath directly into the axillary artery (15) or alternatively through direct percutaneous access of the vessel. However, the vascular complication rate is relatively high with up to 29.2% resulting in endovascular stent-graft implantation due to closure device failure (8).

A new option to facilitate surgical cut-down is a “chimney approach” using an end-to-side anastomosed prosthetic conduit for vessel access (16). This access facilitates the introduction of large self-expanding sheaths into the axillary artery and simplifies the valve mounting maneuver of the balloon-expandable system in the ascending aorta. The chimney approach overcomes access site complications and bleedings from overstretched self-expandable sheaths and is often used for central implanted mechanical circulatory support systems in the case of PAD (17, 18).

We here describe a series of patients treated with TAVR using a TAx approach with a Dacron graft (Terumo Vascular System Corp, Ann Arbor, MI, USA) in combination with a balloon-expandable aortic valve prosthesis.

## Methods

### Patient Population

Between January 2020 and December 2020, 251 patients underwent TAVR at our center (19). In total, 210 patients underwent TF-TAVR (83.7%), 10 patients (3.9%) with severe AS and severe PAD underwent TAVR using the TAx approach, and 31 Patients (12.4%) underwent TA-TAVR due to small subclavian arteries or previous coronary artery bypass graft (CABG). Written informed consent was obtained from each patient following comprehensive assessment and discussion in the multidisciplinary Heart Valve Team meeting and was deemed best managed with TAVR. This retrospective single-center observational study was performed in accordance with the Declaration of Helsinki. The study protocol was approved by the ethics committee of the Faculty of Medicine of the University of Duisburg-Essen (no. 16-6894-BO). All parameters were analyzed anonymously.

Aortic stenosis severity was assessed using transthoracic echocardiography (TTE) according to the joint *European Society of Echocardiography* recommendations (20). Pre-operative imaging was performed in all patients using electrocardiogram-gated multidetector contrast CT angiography. Image analysis, including three-dimensional (3D) reconstructions extending from the aortic annulus to the superficial femoral artery, was performed using *3mensio Structural Heart* software version 9.1 (Pie Medical Imaging, Maastricht, Netherlands).

### Patient Selection

All patients admitted to the department of cardiology were primarily screened for femoral accessibility evaluating the planning CT angiogram using 3D reconstructions. Particular attention was paid to the caliber of the femoral arteries, the anatomical relationships of the side branches to the femoral head, the presence and extension of atherosclerotic plaques and calcifications, and the degree and extension of tortuosity. Severe bilateral occlusive PAD of the iliac and femoral arteries with a caliber <5.5 mm was considered as a contraindication for the TF approach. In this case, the TAx access was considered the second-best access route, and the right and left subclavian and axillary arteries were assessed on the planning CT angiograms using 3D reconstructions. Particular attention was paid to the aortic take-off of the subclavian artery, a typical site of atherosclerotic calcific plaque apposition (21). The presence of a patent right or left internal mammary artery to right coronary artery or left anterior descending artery was a contraindication for the use of this access due to the increased risk of vascular complication leading to the potentially lethal acute graft occlusion. Additionally, Doppler ultrasound (DUS) of the subclavian artery was performed visualizing and assessing the axillary portion of the vessel to control for pre- or post-interventional vessel stenosis, vessel occlusion, or local hematoma. Assessment of the proximal subclavicular portion of the vessel was only possible using 3D reconstructions of the vessel. Thereafter, all patients were discussed at a multidisciplinary Heart Valve Team meeting, and the TAx approach was deemed to be the most appropriate management strategy in each case.

### TAVR Procedure and Operative Technique

In all cases, general anesthesia was obtained. Central venous access is obtained *via* the left or right internal jugular vein to place a pacemaker for right atrium pacing. A left-sided 6F femoral arterial sheath was placed for pigtail placement.

**Figure 1:**
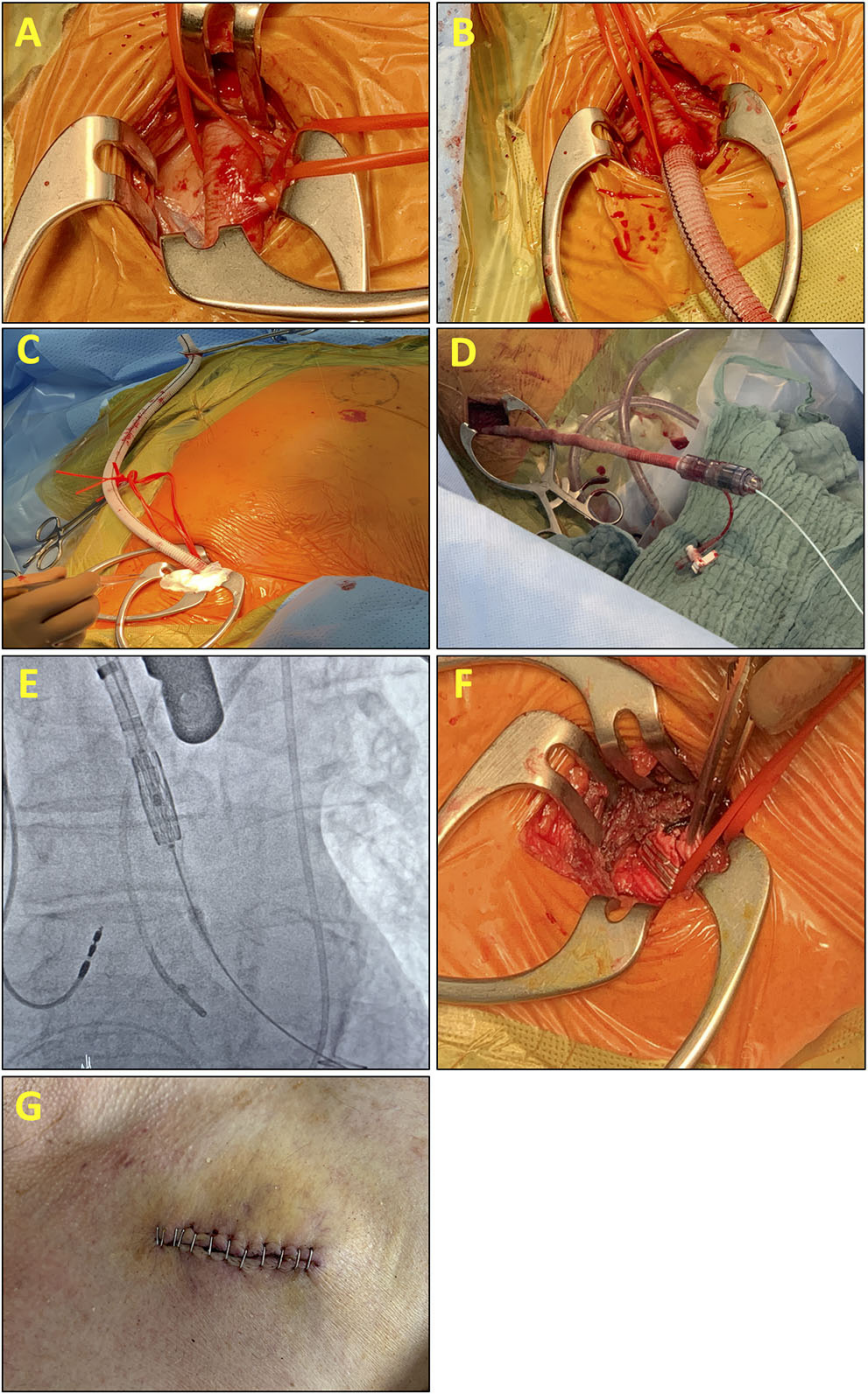
Intraoperative pictures and fluoroscopic images explain the steps of the transaxillary (Tax) end-to-side prosthetic conduit for vessel access. **(A)** Preparation of the axillary artery with **(B)** end-to-side anastomosis of an 8-mm Dacron graft to the axillary artery. **(C)** Final position and length of the Dacron graft before the introduction of the eSheath. **(D)** eSheath is placed through the Dacron graft into the ascending aorta. **(E)** Fluoroscopic-guided advancement of the valve system through the subclavian artery with the Confida wire in the left ventricle. After valve implantation, the e-Sheath is retracted. **(F)** Postinterventional situs: Cut and clipped Dacron graft. **(G)** The wound is closed in a standard fashion with or without a drainage tube according to the preferences of the surgeon.

After detailed skin disinfection, identification of the infraclavicular site and skin incision, the pectoralis minor was divided as required, and the brachial plexus cords were preserved (Figure 1A). The second part of the left or right axillary artery was identified, and proximal and distal controls were obtained. Unfractionated heparin was administered during the procedure. The initial heparin dose was 70 U/kg, and the activated clotting time (ACT) was measured the latest before the insertion of the valve. If not being >250 s, an additional heparin bolus according to body weight was administered.

In nine patients, an 8-mm Dacron graft was anastomosed end-to-side to the axillary artery with a running 6-0 polypropylene suture, leaving the full length of the Dacron graft available to the introducer system (Figure 1B). In one patient, a 10-mm Dacron graft was used (22) (Figure 1C). The 14 French Edwards expandable introducer sheath (eSheath) (Edwards Lifescience Inc., Irvine, CA, USA) guided by a standard 180 cm 0.035 guidewire was inserted in the Dacron graft (Figure 1D). Advancement of the eSheath under fluoroscopic guidance facing the expandable part of the eSheath toward the superior wall of the vessel in line with the axillary artery and the subclavian artery was necessary to avoid increasing trauma to the vessel. The hydrophilic coating of the Edwards introducer system attached itself to the Dacron graft as soon as the complete insertion of the introducer system was finished. Under fluoroscopic guidance, an Amplatzer Left 1 catheter and a straight tipped wire was used to cross the aortic valve. A pigtail catheter was then used to exchange to a Confida Brecker guidewire (Medtronic, Minneapolis, MN, USA) into the left ventricle. Balloon aortic valvuloplasty was not required prior to implant in any patients (Figure 1E).

The technical challenge of deploying an Edwards TAVR *via* the axillary artery is that there is only limited space within the ascending aorta for the preparation of the valve. The critical step is to advance the sheath into the aortic arch just proximal to the entry into the left or right subclavian artery. In three cases with a short ascending aorta, the nose cone of the Commander Delivery System (Edwards Lifescience Inc., Irvine, CA, USA) was passed through the aortic valve into the left outflow tract. With the nose cone beyond the aortic annular plane, it is important to keep the delivery sheath in place to prevent the valve system from moving further into the left ventricle increasing the risk for ventricular rupture by a guidewire or nose cone displacement. Thereafter, the valve system is mounted as described in the instruction for use (IFU) of the Edwards Valve System. Mounting the valve system must be done quickly to prevent prolonged aortic regurgitation from worsening hemodynamics. Finally, the assembly is advanced together into the deployment position. The right sided axillary approach is technical even more challenging due to steeper angle between the subclavian artery and the ascending aorta compared to the left subclavian artery. Additionally, the distance from the ostium of the subclavian artery to the annular plane is shorter leading to increasing the risk of ventricular rupture and worsening hemodynamics due to prolonged mounting maneuvers.

When satisfactory positioning was achieved, rapid pacing was initiated, and the valve is deployed using the identical technique as that during routine implantation *via* the femoral artery. After valve implantation, the delivery system is withdrawn into the sheath and an angiogram is taken to confirm the correct positioning of the valve. A transthoracic echocardiogram was used to assess hemodynamic parameters (**Table 2**). The delivery system was then removed from the body under fluoroscopic guidance (Figure 1F). At the end of the procedure, the Dacron graft was clipped close to the subclavian artery, cut-off just distally of the clip, and the cut was sewn over (Figure 1G). Tight banding was not necessary.

### Anticoagulation Regime Before and After TAVR

If percutaneous coronary intervention (PCI) was performed before TAVR dual antiplatelet therapy (DAPT) was continued for up to 6 months post-PCI and thereafter reduced to single antiplatelet therapy (SAPT) consisting of aspirin monotherapy lifelong. In patients without previous PCI, a loading dose of clopidogrel (600 mg per os) was administered after completion of the TAVR procedure and continued for 6 months according to 2017 guideline recommendations (20). In patients with the need for oral anticoagulation (OAK) being on vitamin K antagonist (VKA) before TAVR anticoagulation was paused until the International Normalized Ratio (INR) of 2.0 was reached. If necessary, bridging with intravenous (i.v.) full-dose unfractionated heparin (FDUH) was started before TAVR when INR was below 2.0. Heparin was paused 6 h before TAVR. Novel Oral Anticoagulants (NOACs) were stopped at least 48 h before the TAVR and resumed on the day after the procedure. Patients with the need for OAK and PCI before TAVR were continued on OAK and DAPT for 4 weeks. Bridging with FDUH was resumed on the first day after TAVR. VKA was simultaneously started. NOAC was re-initiated on the first post-operative day if the access site was uneventful. Thereafter, the anticoagulation regime was reduced to lifelong OAK and single platelet inhibition for 5 more months.

### Endpoint Definition

Peri- and post-procedural complications were evaluated according to the Valve Academic Research Consortium 3 (VARC-3) (23) (Supplementary Table S1).

### Statistics

All continuous data are reported as a mean, with or without SD. All categorical data are reported as percentages of the group. All statistical analyses were performed with SPSS 27.0.1.0 (IBM, Armonk, NY, USA).

## Results

### Patient Population and Anatomic Data

The mean age was 79.8 ± 4.0 years. The mean aortic pressure gradient was 42.7 ± 20.1 mmHg, and the pre-procedural calculated aortic valve area was 0.75 ± 0.2 cm^2^. The mean left ventricular ejection fraction was 42.0 ± 10.8% (range: 28–60%), the logistic European System for Cardiac Operative Risk Evaluation (EuroSCORE) was 18.4 ± 9.7% (STS Score 4.3 ± 2.4%), and 90% of the patients were in New York Heart Association functional class III or IV. Patient characteristics are summarized in Table 1.

**Table 1:** Baseline characteristics of the study group.

**Variables**	**Overall (*n* = 10)**
Age (years)	79.9 ± 4.0
Male patients	7 (70)
Body mass index (kg/m^2^)	24.9 ± 4.8
NYHA III/IV	9 (90)
Coronary artery disease	9 (90)
Prior coronary artery bypass graft	0
Prior percutaneous coronary intervention	6 (60)
Atrial fibrillation	5 (50)
Previous cerebrovascular event	1 (10)
Peripheral vascular disease prohibiting TF-TAVR	10 (100)
Cerebral vascular disease	1 (10)
Diabetes mellitus	26 (31.3)
Renal insufficiency (GFR <60 ml/min/m^2^)	5 (50)
GFR (ml/min/^2^)	54.5 ± 24.3
Logistic EuroScore (%)	18.4 ± 9.7
EuroScore II (%)	5.5 ± 4.0
Society of thoracic surgeons score (%)	4.3 ± 2.4
Left ventricular ejection fraction (%)	42.0 ± 10.8
Aortic valve area (cm^2^)	0.75 ± 0.2
Mean aortic pressure gradient (mmHg)	42.7 ± 20.1
Mean diameter axillary artery (mm)	6.7 ± 0.79

*Data are presented as mean ± SD or number (%); NYHA, New York Heart Association; GFR, glomerular filtration rate; TF-TAVR, transfemoral TAVR*.

### Procedural Success and 30-Day Major Adverse Cardiac and Cerebrovascular Events

Transcatheter aortic valve replacement was performed using the left axillary artery in nine patients. In one case, the right axillary artery was used. The mean diameter of the axillary arteries was 6.7 ± 0.8 mm. In nine patients, an 8-mm Dacron graft was used to match with the 14F and 16F Edwards eSheath, respectively. In one patient, a 10-mm Dacron graft was used. In this case, a thick silk suture was needed to prevent blood loss from the distal part of the graft. Calcification was absent in eight patients, one patient had mild calcification, severe calcification was present in one patient, respectively. DUS was performed before TAVI procedure and confirmed pre-interventional computed tomography (CT) findings. However, assessment of vessel calcification with DUS was not possible in the proximal subclavicular portion of the subclavian artery.

The incision-suture time was 91 ± 36 min (range 49–169 min). TAVR procedure time was 34 ± 16 min (range 15–34 min). Procedural device success according to the Valve Academic Research Consortium (VARC-3) criteria (23) was achieved in all patients (Table 2). Conversion to open-heart surgery was not necessary for any patient.

**Table 2:** Procedural details and adverse events.

**Variables**	**Overall *n* = 10**
Device success	10 (100)
Incision-suture time (min)	91.5 ± 36.3
Procedure time TAVR (min)	34.5 ± 16.3
Fluoroscopy time (min)	8.0 ± 1.5
Contrast (ml)	116.0 ± 39.8
Mean aortic pressure gradient post-TAVR (mmHg)	11.5 ± 4.2
Length of postoperative hospital stay (days)	9.2 ± 6.4
Total hospital stay (days)	18.8 ± 9.0
Conscious sedation	0
Prior valvuloplasty	0
Annular rupture	0
Coronary obstruction	0
Valve size edwards sapien 3 (mm)	
23	2
26	6
29	2
New permanent pacemaker	1 (10)
*VARC-3 complications*	
VARC-3 bleeding complications (BARC-Bleeding complications)	
Type 1 (BARC 2)	2 (20)
Type 2 (BARC 3a)	0
Type 3 (BARC 3b, 3c)	0
Type 4 (BARC 5a, 5b)	0
VARC-3 vascular complications	
Minor	1 (10)
Major	0
Periprocedural severe fatal Stroke (VARC-3)	1 (10)

*Data are presented as mean ± SD or number (%); NYHA, New York Heart Association; GFR, Glomerular filtration rate; BARC, Bleeding Academic Research Consortium; VARC-3, Valve Academic Research Consortium*.

Obstruction of the coronary arteries by the valve prosthesis was not observed. The invasive mean postprocedural aortic transvalvular gradient was 11.5 ± 4.2 mmHg. Mild postprocedural aortic regurgitation was present in two patients (20%), trivial or no aortic regurgitation was seen in eight patients (80%). Periprocedural fatal stroke occurred in one patient (10%). The patient was presented with severe calcification of the left subclavian ostium, calcification of the aortic arch, and plaque of both carotid arteries. TAVR-access site was the LSA. Postinterventional CT showed ischemic infarction in the territory of the anterior cerebral artery and in the left posterior cerebellar artery with subsequent hemiplegia of the left hand. This patient had subsequently died 24 d later due to severe respiratory insufficiency based on severe pneumonia. Two bleeding complications (VARC-3 Type 2) were detected in patients on OAK. Bleedings were located at the cut-down site leading to minor vascular complications (VARC-3 minor) and could be handled in both patients conservatively (20%). No blood transfusions due to bleeding complications were necessary (Table 2). No ischemic complication due to distal thromboembolism was detected.

Doppler ultrasound before discharge showed no stenosis or occlusion of axillary arteries in any patient. Permanent pacemaker implantation due to new onset of complete or high-grade atrioventricular was necessary for one patient (10%).

## Discussion

This case series describes the first-time procedural steps and postprocedural results of TAVR with new-generation balloon-expandable valves using a surgical cut-down and a prosthetic conduit (“chimney approach”) for axillary artery access.

The use of TAx TAVR is well-known for years and was described in 2008 for the first time (15). Since then, several technical improvements and increased operator familiarity with the method contributed to making this approach the second choice in many TAVR centers (24). Most subclavian registries, describing the subclavian approach, were technically TAx given the infraclavicular approach. The largest study to report TAx access with balloon-expandable valves was a single-center experience, including 100 cases of various valve types (25). Only limited case reports have been published using the newest generation balloon-expandable platform, the SAPIEN 3 Ultra (Edwards Lifescience, Irvine, California, USA), from a TAx approach (9, 26).

The end-to-side anastomosis of a Dacron vascular graft was described previously only using self-expandable second-generation valves (16). Some studies are promoting the completely percutaneous use of the TAx-TAVR technique. However, implantation rates of covered stents due to vascular complications or insufficient closure with vascular closure devices are observed in up to 50% of the patients, promoting further stent-related complications and driving interventional costs (24, 26–28).

We started to combine TAx surgical cut-down and end-to-side anastomosis of a Dacron vascular graft to facilitate save vessel access and valve preparation of the Edwards balloon-expandable valves in the ascending aorta. This modified technique avoids extensive manipulation of the artery in case of borderline vascular diameter allowing safe implantation even in patients with patent left or right internal mammary artery to the left anterior descending or right coronary artery compared to the direct open axillary access.

Transaxillary access was applied in only 3.9% of our TAVI population. This is in contrast to previous studies using the TAx approach in 5–10% of the cases when TF TAVR is not feasible (29). Considering the high proportion of patients who underwent TA-TAVR (12.4%) in our center and considering the necessity of thoracotomy leading to delayed mobilization and prolonged hospitalization, increased use of the TAx access seems to be reasonable.

The end-to-side anastomosis of a vascular graft allows prolonged manipulation of large sheaths inside the axillary and subclavian artery and completely accommodates the expandable Edwards eSheath (Edwards Lifescience Inc., Irvine, CA, USA) in different sizes outside the body in the Dacron graft simplifying the valve mounting maneuver of the Edwards Sapien S3 valve in the ascending aorta. Additionally, it is possible to keep the introducer sheath of the TAVR system above the aortic valve due to the “concertina effect” of the Dacron graft while hosting the eSheath of the Edwards Valve System. Applying this combination of Dacron vascular end-to-side graft with surgical cut-down and new-generation balloon-expandable valves led to a 100% implantation success rate.

The TAx approach is routinely used for other vascular interventions, such as complex aortic pathology with fenestrated endografts and extra-anatomic bypasses, while other traditional upper extremity access routes, such as the brachial artery, have problems due to sheath size limitations or frequent complications, such as thrombosis and risk of peripheral neurologic deficits (30). In this series, we were able to show a low peri- and post-interventional access site complication rate. We performed DUS to assess pre- and post-interventional vessel states. Compared to CT, DUS is radiation free and does not expose the patient to contrast agents. Hereby, we could exclude any access site stenosis, vessel occlusion, or vessel thrombosis. Two minor cut-down site bleeding complications (VARC-3 Type 2 bleeding) were detected in our cohort in patients on OAK and could be treated conservatively by tight compression bandage. No blood transfusions due to bleeding complications were necessary. This contrasts with other studies promoting a complete percutaneous approach. These studies documented higher major access site complications ranging from 14 to 30% (25, 27). Postinterventional monitoring is particularly important. In studies describing direct percutaneous access, stent rate is high to resolve access site complications, such as bleeding with long-term stent complications, i.e., deformation and stent thrombosis in up to 18%.

Specific local access site complications described before, such as brachial plexus injury due to the axillary approach at the deltopectoral groove, could not be found in our series. This is in line with other studies suggesting low peripheral neurological complications (27, 31). The rate of periprocedural stroke is significantly higher in patients receiving TAVR through a TAx approach compared to the TF approach as described in a meta-analysis (OR 1.53 (95% CI, 1.05–2.22) (26, 32). However, these studies included only patients where the TAx approach was performed through a direct surgical cut-down without Dacron end-to-side graft. We observed one fatal stroke in a patient with severe calcification of the aortic arch and the ostium of the LSA. This is in line with previous studies emphasizing the need to identify the anatomic characteristics, such as severe calcifications of the axillary artery, the proximal part of the subclavian artery, and the aortic arch, which may lead to embolization of atheromatous plaque during the sheath transfer into the ascending aorta (32).

Bleeding control during the intervention and before TAVR positioning is of paramount importance for surgical access. Unfractionated heparin with an initial bolus of 5,000 IE units and an additional bolus according to weight were administered during the procedure to achieve an ACT target >250 s. Normalization of peri-interventional heparin anticoagulation with protamine was not necessary. Insertion of a drain because of peri-interventional bleeding was not necessary for any patient. Surgical site infection is an ever-present danger. To tackle this issue, all procedures must be performed under sterile conditions. In our study, no access site infection was observed. Therefore, mobilization of patients was possible on the next day after intervention with the goal to keep postinterventional hospitalization as short as possible. Postinterventional length of hospitalization was 9.2 ± 6.4 d, ranging from 4 to 24 days. This seems to be higher compared to other studies. However, our patient cohort includes urgent inpatients in whom complete pre-TAVR screening was performed and a postinterventional rehabilitation facility or a nursing home was to be organized during the hospitalization. However, our study group is rather small to draw definitive conclusions.

### Study Limitations

The present case series has several limitations that should be acknowledged. Most of the patients qualify for TF-TAVR. Therefore, the sample size is relatively limited (3.9%) and a larger series may improve technique and results. Additionally, multiple patients did not present at the outpatient clinic at 3-month or 1-year follow-up, resulting in an inability to report on VARC-3 adverse events beyond 30 days.

## Conclusion

In patients with high or prohibitive risk and no suitable femoral access site, TAx-TAVR using the end-to-side anastomosis of a prosthetic conduit offers a valuable alternative to TF-TAVR after a detailed evaluation of the axillary anatomy.

## Data Availability Statement

The original contributions presented in the study are included in the article/Supplementary Material, further inquiries can be directed to the corresponding author.

## Ethics Statement

The studies involving human participants were reviewed and approved by Ethics Committee of the Faculty of Medicine of the University of Duisburg-Essen. The patients/participants provided their written informed consent to participate in this study.

## Author Contributions

AL, FA-R, and RJ: conceptualization and writing—original draft preparation. AL, AZ, and FA-R: formal analysis. TR and AR: data curation. AL, RJ, MT, and FA-R: writing—review and editing. All authors have read and agreed to the published version of the manuscript.

## Conflict of Interest

The authors declare that the research was conducted in the absence of any commercial or financial relationships that could be construed as a potential conflict of interest.

## Publisher's Note

All claims expressed in this article are solely those of the authors and do not necessarily represent those of their affiliated organizations, or those of the publisher, the editors and the reviewers. Any product that may be evaluated in this article, or claim that may be made by its manufacturer, is not guaranteed or endorsed by the publisher.
