# Supplementary material for: The Transaxillary Approach via Prosthetic Conduit for Transcatheter Aortic Valve Replacement With the New-Generation Balloon-Expandable Valves in Patients With Severe Peripheral Artery Disease
Source: Front Cardiovasc Med. 2022 Jan 13;8:795263. doi: 10.3389/fcvm.2021.795263 (PMC8793794; doi:10.3389/fcvm.2021.795263)
Supplement: Supplementary file 1 [file Table_1.docx]

**Supplemental Material**

Table 1

VARC-3 Criteria

**TIA and Stroke**

| **All Stroke** | Ischaemic stroke  Acute onset of focal neurological signs or symptoms conforming to a focal or multifocal vascular territory within the brain, spinal cord, or retina (NeuroARC Type 1a or 1aH) and fulﬁlling one of the following criteria:   - Signs or symptoms lasting ≥24 h or until death, with pathology or neuroimaging evidence of CNS infarction, or absence of other apparent causes - Symptoms lasting <24 h, with pathology or neuroimaging conﬁrmation of CNS infarction in the corresponding vascular territory   Haemorrhagic stroke  Acute onset of neurological signs or symptoms due to intracranial bleeding from intracerebral or subarachnoid haemorrhage not due to trauma (NeuroARC Types 1b or 1c)  Stroke, not otherwise speciﬁed  Acute onset of neurological signs or symptoms persisting ≥24 h or until death but without sufﬁcient neuroimaging or pathology evidence to be classiﬁed (NeuroARC Type 1d) |
| --- | --- |
|  | TIA  Transient focal neurological signs or symptoms lasting <24 h presumed to be due to focal brain, spinal cord, or retinal ischaemia, but without evidence of acute infarction by neuroimaging or pathology, or with no imaging performed (NeuroARC Type 3a or Type 3aH) |
|  | Delirium without CNS injury  Transient non-focal neurological signs or symptoms, typically of variable duration, without evidence of infarction on neuroimaging or pathology, or with no imaging performed (NeuroARC Type 3b) |
| **Stroke Grading** | |
| Acute stroke severity | - Mild neurological dysfunction: NIHSS 0-5 - Moderate neurological dysfunction: NIHSS 6-14 - Severe neurological dysfunction: NIHSS ≥15 |
| Stroke Disability | - Fatal Stroke: death resulting from a stroke - Stroke with disability: mRS score of ≥2 at 90 days and increase of ≥1 from pre-stroke baseline - Stroke without disability: mRS score of 0 (no symptoms) or 1 (able to carry out all usual duties and activities) at 90 days or no increase in mRS category from pre-stroke baseline |
| Neurological events timing | - Periprocedural: Occurring ≤30 days after the index procedure - Acute: Occurring ≤24 h after the index procedure - Sub-acute: Occurring >24 h and ≤30 days after the index procedure - Early: Occurring >30 days and ≤1 year after the index procedure - Late: Occurring >1 year after the index procedure |

**Bleeding**

| Type 1 | - Overt bleeding that does not require surgical or percutaneous intervention, but does require medical intervention by a health care professional, leading to hospitalization, an increased level of care, or medical evaluation (BARC 2) - Overt bleeding that requires a transfusion of 1 unit of whole blood/red blood cells (BARC 3a) |
| --- | --- |
| Type 2 | - Overt bleeding that requires a transfusion of 2–4 units of whole blood/red blood cells (BARC 3a) - Overt bleeding associated with a haemoglobin drop of >3 g/L (>1.86 mmol/L) but <5 g/d (<3.1 mmol/L) (BARC 3a) |
| Type 3 | - Overt bleeding in a critical organ, such as intracranial, intraspinal, intraocular, pericardial (associated with haemodynamic compromise/ tamponade and necessitating intervention), or intramuscular with compartment syndrome (BARC 3b, BARC 3c) Overt bleeding causing hypovolemic shock or severe hypotension (systolic blood pressure <90 mmHg lasting >30 min and not responding to volume resuscitation) or requiring vasopressors or surgery (BARC 3b) - Overt bleeding requiring reoperation, surgical exploration, or reintervention for the purpose of controlling bleeding (BARC 3b, BARC 4) - Post-thoracotomy chest tube output ≥2 L within a 24-h period (BARC 4) - Overt bleeding requiring a transfusion of ≥5 units of whole blood/red blood cells (BARC 3a) - Overt bleeding associated with a haemoglobin drop ≥5 g/dL (≥3.1 mmol/L) (BARC 3b). |
| Type 4 | Overt bleeding leading to death. Should be classiﬁed as:   - Probable: Clinical suspicion (BARC 5a) - Deﬁnite: Conﬁrmed by autopsy or imaging (BARC 5b) |

**Acute kidney injury (AKNI classification)**

| Stage 1 | Increase in serum creatinine to 150–199% (1.5–1.99 × increase compared with baseline) OR increase of ≥0.3 mg/dl (≥26.4 mmol/l) OR Urine output <0.5 ml/kg/h for >6 but <12 h |
| --- | --- |
| Stage 2 | Increase in serum creatinine to 200–299% (2.0–2.99 × increase compared with baseline) OR Urine output <0.5 ml/kg/h for >12 but <24 h |
| Stage 3 | Increase in serum creatinine to ≥300% (>3 × increase compared with baseline) OR serum creatinine of ≥4.0 mg/dl (≥354 mmol/l) with an acute increase of at least 0.5 mg/dl (44 mmol/l) OR Urine output <0.3 ml/kg/h for ≥24 h OR Anuria for ≥12 h |

**Vascular access site and access-related complications**

| Major vascular complications | One of the following:   - Aortic dissection or aortic rupture - Vascular (arterial or venous) injury (perforation, rupture, dissection, stenosis, ischaemia, arterial or venous thrombosis including pulmonary embolism, arteriovenous ﬁstula, pseudoaneurysm, haematoma, retroperitoneal haematoma, infection) or compartment syndrome resulting in death, VARC type ≥2 bleeding, limb or visceral ischaemia, or irreversible neurologic impairment - Distal embolization (non-cerebral) from a vascular source resulting in death, amputation, limb or visceral ischaemia, or irreversible end-organ damage - Unplanned endovascular or surgical intervention resulting in death, VARC type ≥2 bleeding, limb or visceral ischaemia, or irreversible neurologic impairment - Closure device failure resulting in death, VARC type ≥2 bleeding, limb or visceral ischaemia, or irreversible neurologic impairment |
| --- | --- |
| Minor vascular complications | One of the following:   - Vascular (arterial or venous) injury (perforation, rupture, dissection, stenosis, ischaemia, arterial or venous thrombosis including pulmonary embolism, arteriovenous ﬁstula, pseudoaneurysm, haematoma, retroperitoneal haematoma, infection) not resulting in death, VARC type ≥2 bleeding, limb or visceral ischaemia, or irreversible neurologic impairment n Distal embolization treated with embolectomy and/or thrombectomy, not resulting in death, amputation, limb or visceral ischaemia, or irreversible end-organ damage - Any unplanned endovascular or surgical intervention, ultra-sound guided compression, or thrombin injection, not resulting in death, VARC type ≥2 bleeding, limb or visceral ischaemia, or irreversible neurologic impairment - Closure device failure not resulting in death, VARC type ≥2 bleeding, limb or visceral ischaemia, or irreversible neurologic impairment |
| Major access-related non-vascular complications | One of the following:   - Non-vascular structure, non-cardiac structure§ perforation, injury, or infection resulting in death, VARC type ≥2 bleeding, irreversible nerve injury or requiring unplanned surgery or percutaneous intervention - Non-vascular access site (e.g. trans-apical left ventricular) perforation, injury, or infection resulting in death, VARC type ≥2 bleeding, irreversible nerve injury or requiring unplanned surgery or percutaneous intervention |
| Minor access-related non-vascular complications | One of the following:   - Non-vascular structure, non-cardiac structure perforation, injury, or infection not resulting in death, VARC type ≥2, irreversible nerve injury, or requiring unplanned surgery or percutaneous intervention - Non-vascular access site (e.g. trans-apical left ventricular) perforation, injury, or infection not resulting in death, VARC type ≥2 bleeding, irreversible nerveinjury or requiring unplanned surgery or percutaneous intervention |
